# Effects of Selective Neonatal Amygdala Damage on Concurrent Discrimination Learning and Reinforcer Devaluation in Monkeys

**DOI:** 10.4172/2161-0487.S7-005

**Published:** 2013-08-27

**Authors:** AM Kazama, J Bachevalier

**Affiliations:** Yerkes National Primate Research Center and Department of Psychology, Emory University, Atlanta, GA, USA

## Abstract

**Objectives:**

The amygdala is known to be a key neural structure in many neuropsychiatric disorders. Primarily known for its involvement in fear regulation, the amygdala also plays a critical role in appetitive flexible decision-making. Yet, its contribution to the development of flexible goal-directed behavior has not been thoroughly examined.

**Design:**

The current study examined flexible decision-making abilities after neonatal amygdala lesions in nonhuman primates using a behavioral paradigm known to measure the flexible monitoring of goal-directed choices in rodents, monkeys, and humans.

**Method:**

Rhesus monkeys of both sexes were divided into two groups, a sham-operated control group (N=4) and a group with neonatal neurotoxic amygdala lesions (N=5). Animals received the lesions at 1–2 weeks and were tested at both four and six years of age on a concurrent discrimination reinforcer devaluation task.

**Results:**

Although neonatal amygdala damage spared learning stimulus-reward associations, it severely impaired the ability to flexibly shift object choices away from those items associated with devalued food rewards. The results were similar to those obtained in monkeys that had acquired the same lesions in adulthood.

**Conclusions:**

Thus, the amygdala is critical for appetitive decision-making, and provide further evidence of little functional sparing after early amygdala insult. The findings are discussed in relation to other behavioral measures on the same animals and to clinical neuropsychiatric disorders.

## Introduction

A growing trend in neuropsychiatric research is the transition from relying primarily on behavioral symptom-based diagnoses [1] to include biomarkers (endophenotypes) as a clinical basis for diagnosis [2,3]. Thus, a critical need for basic science is to identify and characterize the behavioral contributions of key neural structures found to be dysfunctional in specific psychopathology. For example, amygdala dysfunction has been identified in many neuropsychiatric disorders including classic developmental disorders such as Autism [4], William’s Syndrome [5], and Schizophrenia [6,7], Post-Traumatic Stress Disorder (PTSD) [8], and other anxiety disorders [9]. Interestingly, these syndromes are now thought to have a strong developmental component [10]. Thus, understanding not only the basic functions of the amygdala, but also how its early perturbation may lead to specific behavioral deficits, which may or may not differ depending on the type of pathologic insult and/or the age at which the insult occurs, is hugely relevant for providing clinically relevant biomarkers and determining optimal treatment options.

To increase our knowledge in this area, our approach combined early damage to selective nodes in the neural network supporting decision-making with neuropsychological investigations across development [11]. A non-human primate animal model was used to examine the long-term consequences of selective damage to the amygdala, received either in infancy or in adulthood, across many behavioral and cognitive dimensions, including socio-emotional behavior [12], hormonal regulation [13–15], regulation of fear [13,16,17], and cognitive/behavioral flexibility [18–20]. The present paper will more specifically inform on the contribution of the amygdala to the development of flexible regulation of goal-directed behaviors.

In a recent study [16], we investigated the long-term outcomes of early amygdala insult in the development of safety-signal learning, a known biomarker for PTSD [21]. Using the AX+BX- paradigm [22], we found that early amygdala damage resulted in the classic impairment in fear learning. However, once animals learned to discriminate between fear and safety signal cues, they were able to flexibly use this information to regulate their emotion as efficiently as control animals. Thus, the sparing of conditioned inhibition after neonatal amygdala lesions clearly challenged the amygdalocentric model of the fear response, which serves as the basis for models of PTSD and other anxiety disorders [23]. It also suggests that the modulation of fear by contextual signals may rely on brain areas processing safety signals and exerting their modulation via connections to areas other than the amygdala along the startle pathway. This then begged the question: would similar sparing of function be evident for the modulation of appetitive or rewarding outcomes?

Although, primarily known for its involvement in fear regulation, the amygdala also plays a critical role in appetitive flexible decision-making [24] as defined by the ability to evaluate and choose from multiple behavioral response options that may vary in terms of 1) the magnitude of reward and punishment, 2) the likelihood of receiving the reward or punishment, and 3) the expected delay to receive the reward or punishment [25]. Damage to the amygdala spares certain types of basic behavioral flexibility, such as the ability to form and break stimulus-reward associations as measured by the Object Reversal Task [18,26,27], but severely impacts the flexible updating of those associations provided by external or internal body signals as needed for the Reinforcer Devaluation Task [20,28,29]. However, this deficit in the modulation of goal-directed behaviors was observed when the lesions were acquired in adulthood and little is known on whether a similar outcome will follow damage to the amygdala inflicted early during development. Given the sparing of fear regulation after neonatal amygdala lesions reported above, one would predict that the same will hold true for the flexible modulation of goal-directed behaviors. The current study tested this proposal by examining the effects of neonatal amygdala lesions in the development of appetitive flexible decision-making abilities, using a translational reinforcer devaluation paradigm that has been employed in humans [30], rodents [31,32], and in monkeys [19,33]. Preliminary data have already been published in an abstract form [34,35].

## Methods

### Subjects

Ten rhesus macaques (*Macaca mulatta*) of both sexes and ranging from 4.5 – 8 kg participated in this study at approximately 3–4 years and 5–6 years of age. All animals had received surgical brain procedures between 8–12 days of age which included either neurotoxic lesions of the amygdala (Group Neo-Aibo, 3 males and 3 females) or sham-operations (Group Neo-C, 2 males, 2 females). The Animal Care and Use Committees of the University of Texas Health Science Center at Houston and of Emory University approved all of the following procedures.

Rearing conditions have been described previously [36], thus a brief description is provided below. Animals were housed individually in small wire-cages, maintained on a 12 hour light/dark cycle, and given extensive social peer contact as well as contact with human caregivers every day. At one year of age, animals were moved into larger enclosures, housing four animals and allowing permanent social contacts with peers. Animals were provided age appropriate diets of Similac (SMA with Iron) at 0–3 months, which was supplemented with banana-flavored pellets (PJ Noyes, Cleveland, OH). From 3–12 months, animals were introduced to Purina primate chow (Purina, St. Loyis, MI) and fresh fruit, which was continued in adulthood. Water was given *ad libitum* starting at three months of age.

All monkeys received behavioral testing prior to being used in this study as well as between the two ages during which the discrimination/devaluation task was given. This training included tasks measuring recognition and relational memory abilities (Bachevalier, unpublished data), object discrimination reversal [37], emotional responses to fearful stimuli [15], social attachment [36], and social interactions [38].

### Neuroimaging and surgical procedures

Detailed descriptions are provided in a previous report [36]. Briefly, animals were first anesthetized using isoflurane gas (1–2% to effect), and given an intravenous drop solution (0.45% NaCl) to maintain hydration. Vital signs (heart rate, respiration, blood pressure, body temperature, and expired CO_2_) were monitored during all procedures and body temperature was maintained with warm blankets surrounding the animal. The animal’s head was then secured in a non-ferric stereotaxic apparatus, and imaged using a GE signa 1.5 Tesla Echo Speed scanner and a 7.5 cm circular head coil (GE Medical Systems, Milwaukee, WI). The pre-surgical brain imaging included two sequences [12]: a 3D T1-weighted fast spoiled gradient (FSPGR)-echo 1-mm images obtained in the coronal plan that was used to precisely visualize the location of the amygdala and select the injection sites, followed by three series of 3-mm Fluid Attenuated Inversion Recovery coronal images offset of 1 mm in the anterior-posteriorly axis. The same sequences were repeated for animals in group Neo-Aibo 7–8 days after surgery and the images were used to assess lesion extent.

Following the pre-surgical MRI scans, the animals were kept anesthetized and brought to the surgical suite where the surgical procedures were performed using aseptic techniques. The skin on the scalp was disinfected and a local anesthetic (Marcaine 25%, 1.5 m., s.c.) was injected along the midline skin incision to reduce pain. After retraction of the conjunctive tissue, a small craniotomy was performed in both hemispheres, just above the amygdala, and the dura was cut to expose the brain. For sham-operations, the procedure stopped at this point. For the neurotoxic amygdala lesions, 4–6 sites (2mm apart in all directions) were selected to target the center of the amygdala and 0.2–0.6 µl of ibotenic acid (Biosearch Technologies, Novato, CA, 10mg/ml in phosphate buffered saline, pH 7.4) was injected at each site at a rate of 0.4 µl/min using 10 µl Hamilton syringe held on a Kopf electrode manipulator (David Kopf Instruments, Tujunga, CA). Injections were simultaneously done in both hemispheres.

After the surgical procedures, the openings were sutured in anatomical layers. The animals were then brought back to consciousness and allowed to recover in an incubator ventilated with oxygen. Antibiotic (Cephazolin, 25 mg/kg, per os) and anti-inflammatory (dexamethazone sodium phosphate, 0.4 mg/kg, s.c.) were started 12 hours prior to surgery and were continued until post-surgical day seven. Additionally, to relieve pain, a topical antibiotic ointment/anesthetic was applied on the wound and Acetaminophen (10mg/kg, p.o.) were administered four times a day for three days after surgery.

### Lesion verification

The pre- and post-surgical FLAIR images were used to identify areas of hypersignals (indicative of brain edema caused by cell death) and were compared to the FSPGR images to accurately identify the borders of the brain structures. Extent of hypersignals seen on post-surgical FLAIR images at 1 mm intervals was drawn onto matched coronal sections of a normal infant rhesus macaque brain (J. Bachevalier, unpublished data). These drawings were then imported into ImageJ® (available at http://rsb.info.nih.gov/ij; developed by Wayne Rasband, National Institutes of Health, Bethesda, MD) to measure the cross-sectional area (in square pixels) of damage to intended (amygdala) and unintended (entorhinal and perirhinal cortex, and anterior hippocampus) brain areas. The total volume of damage for a given area in each hemisphere was determined by summing the area damaged on each section, and multiplying this sum by slice thickness (1 mm). The volume of damage was then divided by the normal volume of this area, measured on the brain template, to estimate a percentage of the total volume damaged for each brain area [16,36].

### Behavioral paradigm

Animals were tested at 3 – 4 years of age and re-tested at 5 – 6 years using the exact same stimuli. Behavioral testing in the reinforcer devaluation task was similar to methods described to test monkeys that had received similar brain lesions in adulthood [19,29].

#### Apparatus and stimuli

Testing took place in a darkened room equipped with a white noise generator and containing a Wisconsin General Testing Apparatus (WGTA). The apparatus was fitted with a tray holding three food wells, spaced 13 cm apart and aligned in the center of the tray. Only the two lateral wells were used during testing. One hundred-twenty objects, previously selected to test adult monkeys were used. These objects were paired to form 60 easily discriminable pairs matched for size and shape. Within each pair, one object (S+1 or S+2) was always placed above a lateral well containing a food reward (either a peanut, a raisin, or a banana flavored pellet) based on that individual monkey’s past preference with food reward in other behavioral tasks. The other object of the pairs (S−) was placed over the other lateral empty food well.

#### Phase I - concurrent discrimination learning

On each trial, the two lateral wells were covered with the S+ item hiding the reward and the unrewarded object (S−) of the pair. The 60 pairs of objects were presented one at a time at 30-s intervals (i.e. 60 trials per day, 30 with S+1 objects and 30 with S+2 objects intermixed), and given every day until the animal reached a criterion of 90 correct responses in 5 consecutive days of testing. The order of presentation of each pair was kept constant across training but the left-right position of the stimuli varied pseudo randomly [39]. Total sessions and errors to criterion measured rate of acquisition of the 60 problems and scores on first day of acquisition assessed any initial bias toward the S+1 or S+2 stimuli. In addition, daily scores were broken down by food type in order to determine whether or not learning rates might differ depending upon food preferences.

#### Phase II - reinforcer devaluation

Animals were then presented with four test sessions occurring on four separate test days. For these test sessions, the rewarded objects of Phase I (S+1 and S+2) were paired (e.g. S+peanut against S+raisin) to form thirty trials. The S+ pairs and their respective presentation order were kept constant throughout all four sessions, but their left/right positions were varied pseudo randomly each day. The four test sessions were as follows:

#### Baseline I

All 30 pairs were presented consecutively at 30-sec intervals.

#### Devaluation I

Before testing, each animal was given 100 grams of the 1st preferred food reward in its home cage and allowed to eat freely for thirty minutes. Additional food was given every fifteen minutes until five minutes passed without further ingestion of the food by the animal (satiation). Immediately after reaching satiation, the animal was transferred to the WGTA and tested as in Baseline I with the 30 pairs of S+ objects.

#### Baseline II

Same as Baseline I.

#### Devaluation II

The procedures for this test were identical to the Devaluation I, substituting the 2nd food reward in place of the 1st food reward for the satiation procedure.

To ensure that the effects of the reinforcer devaluation conditions did not carry over from one day to the next, one regular 60-trial stage I training session followed each of the baseline sessions, and two days of rest followed each reinforcer devaluation session, followed by a regular training session. For each session, number of S+1 and S+2 objects selected by the animals were recorded as well as whether or not the reward was ingested or discarded by the monkeys.

Several measures were taken to ensure that this experiment was balanced and consistent with previous experiments [19,28,29]. First, the testing order was generated randomly, but counter-balanced across groups and food type. Second, to ensure that animals remained motivated to perform this task at a high level of accuracy, food intake was restricted. Weekly weights were taken to ensure that animals’ weights did not drop below 85% of their normal weight. Third, in an effort to control for any effects of circadian rhythm on the animal’s motivation, all testing was counterbalanced for time of day, and both stage I and stage II were conducted at consistent times for each individual animal.

To assess effects of lesion that were potentially independent of flexible decision-making abilities, several measures were taken according to previous studies [19,28,29]: (1) animal weight before each devaluation probe session, (2) total food intake to reach satiation, and (3) time taken to reach satiation. Baseline scores were used to determine object/food preferences. Difference scores were calculated to assess flexible decision-making abilities. For both selection of the objects associated with the satiated food reward, as well as for consumption of the actual food reward, difference scores were calculated by subtracting the sum of the two baseline scores from the sum of the two satiation scores. The object difference scores indicated the degree to which each subject altered their preferred choice of objects, based on satiation (i.e. select the object associated with the non-satiated food). The food difference scores indicated to what degree each subject continued to consume the devalued food after the object was displaced.

### Statistical analyses

#### Phase I

For the concurrent discrimination learning phase at 4 years, one sample t-tests confirmed that all animals started at chance levels (30/60 correct) and Group × Sex ANOVA compared total trials and errors to criterion. When re-tested at 6 years of age, retention of the 60 discrimination pairs was analyzed with a repeated measure ANOVA (Group × Sex × Age) for trials and errors.

#### Phase II

Performance of the baseline tests was analyzed for both ages separately using paired samples t-tests to assess whether or not animals demonstrated a significant preference for items associated with a specific food reward (S+1 or S+2). Repeated measures ANOVAs (Group × Sex × Age) were conducted using the various satiation variables as the independent measures as well as on both optimal object difference scores and food difference scores.

Note that, due to behavioral issues that impacted its learning performance, one animal in Group Neo-Aibo (Neo-Aibo-4) was dropped from all analyses including the factor Age.

Finally, to assess any sparing of functions following the neonatal lesions, scores obtained at 4 years of age were compared to those of adult animals that had received similar neurotoxic amygdala lesions in adulthood and were tested in the same way at 4 years of age [17], using two-way ANOVAs (Group × Time at lesions). Effect sizes are provided in all cases where the data revealed either significant or trend-like differences.

## Results

### Lesion verification

**Figure 1:**
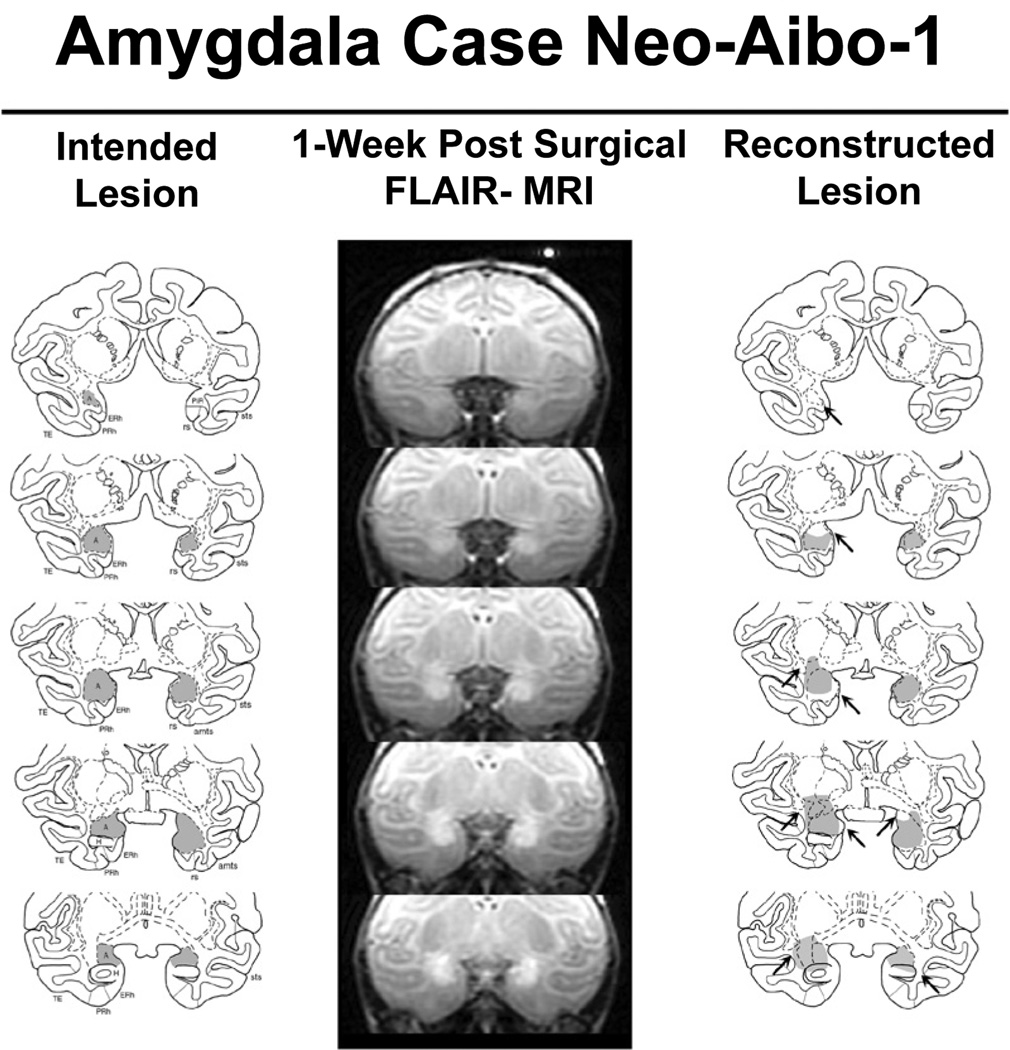
Intended amygdala damage is shown in gray on coronal sections through the amygdala of an infant macaque brain atlas in the left column. Location of hypersignals shown on FLAIR MR coronal images is given at several matched anterior-posterior levels through the amygdala in case Neo-Aibo-1 (middle column). Edema caused by cell death appears white within and around the amygdala. The estimated reconstructed lesion extent is shown in the right column. Arrows point to areas of unintended damage or sparing. Abbreviations: Is – lateral sulcus; sts – superior temporal sulcus; ots – occipital temporal sulcus; ERh – entorhinal cortex; PRh – perirhinal cotex; TE, temporal cortical area and TH/TF – cytoarchitectonic fields of the parahippocampal gyrus.

Extent of amygdala lesions is presented in detail for all cases in table 1, and figure 1 displays a representative case.

Bilateral amygdala damage averaged 62.5%. For three cases (Neo-Aibo −1, −4, and −6), the damage was extensive and symmetrical (from 63.8% to 76% bilaterally) and for the remaining three cases (Neo-Aibo −2, −3, and −5), the damage was more extensive in the right hemisphere (61.1% to 77.6%) than in the left hemisphere (33.0% to 42.0%). In all cases, damage included the medial, central, accessory basal and dorsal areas of the basal nuclei and the majority of sparing was located in the lateral nuclei. The degree of unintended damage to entorhinal/perirhinal cortical areas, anterior hippocampus, and tail of the putamen were non-significant in all cases.

### Phase I: Concurrent discrimination learning

#### Acquisition at 4 years

Both groups performed at chance during the initial 60-trials session (t=−0.785, p>0.05), indicating no significant initial bias associated with external cues or individual object preference for both groups. All animals reached the learning criterion (90% correct over five sessions) within the limit of testing ([Group: F(1, 8)=0.008 and 0.594, ps>0.05, for trials and errors, respectively, see Table 2]. There were no significant Sex effects [F(1, 6)=0.006 and 0.118, *ps*>0.05, respectively], and no significant Group by Sex interactions [*F*(1,6)=0.006 and 0.006,*ps*>0.05, respectively].

#### Retention at 6 years

When re-tested two years later using the exact same stimuli, all animals showed good retention of all stimuli (see Table 2), re-acquiring the task in an average of 61 trials (25 errors) for Group Neo-C and 56 trials (14 errors) for Group Neo-Aibo [Age effects, F (1,7)=20.417, 24.82, p < 0.003, µ^2^=0.75, 0.78, for trials and errors, respectively]. There were no effect of group [F_Huynh-Feldt_ (1,7)=0.023, 0.601, ps>0.05, for trials and errors, respectively] and no significant interactions (all ps>0.05).

### Phase II: Reinforcer devaluation

#### General Satiation Variables (Table 3)

Both groups took the same amount of time to reach satiation criterion with Food #1 [Group: *F*(1, 5)=0.011, p>0.05, Sex: *F*(1, 5)=0.267, p>0.05, all interactions *ps*>0.05] and with Food #2 [Group: *F*(1, 5)=0.35, p>0.05, Sex: *F*(1, 5)=1.11, p>0.05, Age *F*(1, 5)=0.106, p>0.05, all interactions, *ps*>0.05]. Again for amount of Food # 1 and Food # 2 consumed during the satiation, there were no significant effects of Group [*F*(2, 5)=0.041 and 0.614, p>0.05], Sex [*F*(1, 5)=0.142,p>0.05 and 0. 526,p>0.05], or Age [*F*(1, 5)=0.031and 4.907, p>0.05 ], and no significant interactions (all *ps*>0.05).

Finally, as expected, all animals gained approximately a kilogram of body weight [Age: F (1, 5)=21.42, p=0.006], and males weighed more than females with a trend towards significance [Sex: F _Huynh-Feldt_ (1, 5)=5.19, p=0.07, µ^2^=0.51], however the Group effect was not significant [*F*(2,1)=1.62, p>0.05] and none of the interactions reached significance (all ps>0.05).

#### Baseline probe sessions

Paired samples t-tests comparing selection of “S+1 vs S+2” objects for each group at both age revealed that all animals had a significant preference for objects associated with a specific reward during baseline trials (e.g. selection of more peanut items than raisin items) [Age 4: *t*=4.131 and 6.114, *ps* < 0.05, Age 6: *t*=5.29 and 7.27, *ps* < 0.05, for Groups Neo-C and Neo-Aibo, respectively). Thus, both the Group and Age factor did not reach significance [F(1, 7)=0.835, p>0.05 and F(1, 7)=4.435, p>0.05, respectively].

#### Reinforcer devaluation probe sessions

**Figure 2:**
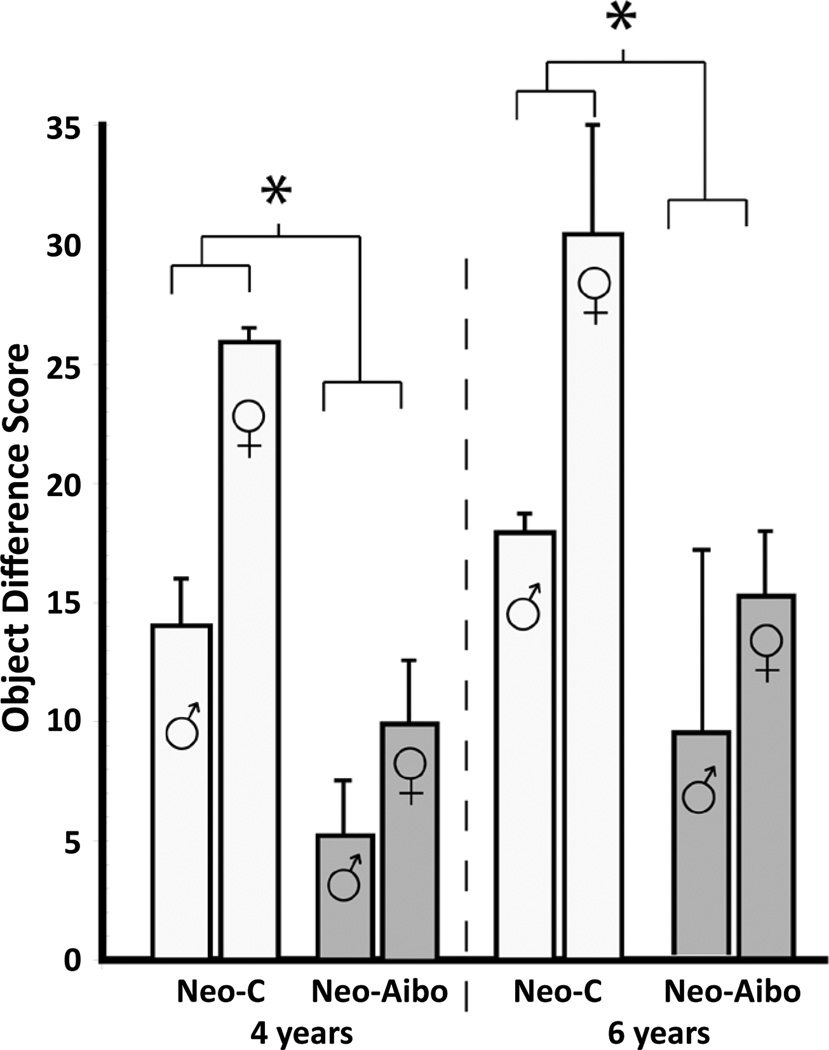
Object difference scores in male and females with neonatal amygdala lesions (Neo-Aibo) and sham-operated controls (Neo-C) at the two ages tested. Object difference score measures how often an animal chooses an object paired with a devalued food item during the satiation probe session as compared to baseline session. Vertical bars provide SEM values and * indicates p < 0.05.

The satiation object difference scores for each animal (Table 2 and Figure 2) were calculated by subtracting the number of objects associated with each food in the baseline sessions and the number objects associated with that same food in the devaluation session when that food had been devalued. Because some animals developed strong food preferences across multiple cognitive tasks involving food rewards, we calculated the optimal devaluation score for each individual animal. Thus, a high object difference score indicates that the animal took more satiated food-related objects during baseline than during the devaluation session, and therefore demonstrated greater flexibility. Although there was a slight improvement across the two time points, a repeated measures ANOVA (Group × Sex × Age) revealed no significant effects of Age [*F*( 1,5)=1.78, p>0.05]. However, as compared to controls, animals with Neo-Aibo lesions demonstrated less flexibility as revealed by lower difference scores [Group: *F*(1, 5)=8.51, p=0.03, µ^2^=0.63]. I*n*terestingly, the data also revealed that females tended to score higher on average than males [M=22.8 vs 13.5, respectively; Sex:*F*(1, 5)=5.48, p=0.07, µ^2^=0.52], but none of the interactions reached significance (all ps>0.05).

The satiation food selection difference scores (see Table 2) measured the number of time the animal actually took and ingested the devalued food after displacing the object. Thus, animals with large food difference scores indicated a refusal to eat the satiated food after the object was displaced. There were again no significant effects of Group [*F*(2, 5)=1.25, p>.05], Sex [*F*(1, 5)=0.683, p>0.05], or Age [*F*(1, 5)=0.279, p>0.05], and no significant interactions [all ps>0.05]. These data confirm that all animals refused satiated food once that food item hadbeen revealed via displacement of the object coveringthe food well.

### Comparisons between the effects of neonatal versus adult lesions

For these analyses, scores obtained for animals with neonatal lesions obtained when they were tested for the first time at 4 years of age were compared to those of animals with adult-onset lesions also tested for the first time at 4 years of age.

#### Phase I- Acquisition (Figure 3)

All animals learned the 60 discrimination problems at the same rate regardless of timing of lesion [Group: F(1, 12)=0.774, p>0.05; Time at lesions: F(1, 12)=0.581, p>0.05; Group × Time at lesions: F(1, 12)=0.581, p>0.05)]. Similar results were obtained for errors to criterion [Group Effect: F(1, 12)=1.02, p>0.05; Time at lesions: F(1, 12)=0.841, p>0.05; Group ×Time at lesions : F(1, 12)=0.016,p>0.05].

#### Phase II- Devaluation (Figure 4)

Damage to the amygdala resulted in significantly less flexible decision-making [Group: F(1, 12)=15.89, p=0.002], regardless of timing of lesions [F(1, 12)=.222, p>.05; Group × Time: F(1, 12)=0.237, p>0.05].

## Discussion

The study revealed several original and clinically relevant findings on the effects of neonatal amygdala lesions on learning reward-contingency and flexible modulation of goal-directed choices by internal body signals. First, learning associations between a visual stimulus and a food reward as well as long-term (2 years) retention of these stimulus-reward associations were unaffected by neonatal damage to the amygdala. Second, despite being aware of which specific food items were associated with specific objects, animals with neonatal amygdala lesions persisted in selecting the devalued S+ items after satiation, indicating an inability to flexibly alter behavioral responses guided by appetitive internal body signals. Interestingly, as compared to males, females tended to have higher reinforcer devaluation scores, but this gender difference occurred in both groups. Finally, a comparison between the effects of early-onset versus adult-onset amygdala lesions revealed no functional sparing after the early lesions.

### Learning stimulus-reward associations

Neonatal damage to the amygdala did not impair the ability to learn concurrent discrimination problems. Additionally, the strong baseline preference scores achieved during the probe test suggests that not only were animals able to quickly form these stimulus-reward associations, but could also remember which food item was associated with specific objects. Animals were also able to retain these associations over a period of two years. This lack of learning impairment is consistent with earlier findings showing that similar neonatal damage did not affect the ability to learn 20 concurrent discrimination problems [40] and 5 concurrent discrimination problems intermixed within a daily session [18], and parallels the normal performance on learning stimulus-associations when damage to the amygdala occurred in adulthood [19,28,29,33,41]. Although the lack of impairment could be due to experience animals gained in cognitive training prior to the present study, the cognitive scores both controls and experimental animals obtained in stimulus-reward association tasks are similar to those obtained by experimentally naïve adult animals tested in the same tasks in earlier reports [28]. The observed sparing lies in sharp contrast to previous fear learning studies using animals that had received their lesions in adulthood [16,42– 44], as well as one study involving these same animals and examining stimulus-fear association abilities as measured by the fear-potentiated AX+/BX- paradigm [16]. Animals were presented with a cue (e.g. light, tone, or fan), which was sometimes paired with an aversive but painless puff of air directed at the animal’s head. Animals with neonatal amygdala damage took six times longer to learn these associations relative to sham-operated controls. Thus, the amygdala appears to be critical for the development of fear learning, but not for simple forms of appetitive learning.

### Reinforcer devaluation

Neonatal lesions of the amygdala affected the animal’s tendency to inhibit selection of objects associated with a devalued reinforcer. This impairment occurred despite animals being able (1) to associate specific stimuli with specific food rewards, as revealed by the tendency to select objects associated with their preferred food more frequently than the objects associated with the other food in the two baseline conditions and (2) to avoid taking the devalued food after displacing the object. These results are consistent with those observed in adult animals that had received neurotoxic lesions [19,28,29,33,41] or transient inactivation [45] of the amygdala in adulthood. The data add to our current knowledge that this function of the amygdala cannot be compensated by other neural substrates even when the lesions of the amygdala occurred in infancy. Nevertheless, what has remained elusive until now is the specific processes by which the amygdala supports performance on the reinforce-devaluation task. Because the animals of the current study had been tested in several tasks measuring flexible goal-directed behaviors or flexible modulation of emotional reactivity and because the neonatal lesions impacted performance on some of these tasks but not others, a fuller discussion of the divergent findings may provide insights on the role of the amygdala in flexible behavioral modulation.

First, the impairment in flexibly altering object selection after food devaluation in animals with neonatal amygdala lesions contrasts with their unimpaired performance on other appetitive tasks measuring behavioral inhibition, such as the object reversal learning tasks (1 pair or 5 pairs, [18]). Although the two tasks measure abilities to modify object selection, there are clear distinctions on the type of information necessary to make the change in selection pattern. As alluded by others [18,19,28], the object reversal task is based on reward contingency in that one object is rewarded and the other is not. When the reward between the two objects is switched without warning, animals must extinguish a previously learned stimulus-reward association and acquire a more appropriate one. By contrast, the reinforcer-devaluation task is based on reward value in that both objects are rewarded but the reward value of one of the two objects has been altered by satiation. The animals must rely on information about changes on their internal state to adjust their response pattern. Given that during the devaluation probe test the animals with neonatal amygdala lesions were avoiding taking the food that had been devalued and hence demonstrated intact discrimination of their internal states [46], impairment in avoiding the objects associated with the sated food may reflect an inability to rapidly modify choice selection based on a single probe trial. Thus, in the absence of a functional amygdala, animals could still rely on other brain structures, such as the orbital frontal cortex and/or the striatum, to slowly modify their choice selection over several trials and demonstrate some flexibility, as required in the object reversal task. By contrast, the ability to rapidly modify choice behavior based on a single (or few exposures), as required in the reinforce devaluation task may require intact amygdala-orbitofrontal interactions [28,33].

A similar dichotomy on the effects of neonatal amygdala lesions was observed on two tasks of emotional modulation. When tested on the AX+/BX− task to measure modulation of startle amplitude when an aversive cue is paired with a safety cue, animals with neonatal amygdala lesions learned the value of each cue (albeit more slowly than controls) and were then able to modulate their reactivity when the two cues were combined demonstrating normal conditioned inhibition [16]. However, when tested in the Human Intruder during which the animals rapidly modulate their emotional responses based on the intensity of threat depicted by the Human Intruder, the same animals reacted to the presence of the Intruder but did not modulate their emotional responses according to threat intensity [14]. Again, the different outcomes of the neonatal amygdala lesions on these two tasks may relate to the knowledge the animals already possess on the environmental cues mediating their behavioral responses. In the absence of a functional amygdala, the valence of the cues in the AX+/BX– task has been well learned, most likely involving other brain structures that could also mediate flexible modulation. For instance, when human subjects learn to associate one cue with a mild shock and a second cue with no shock, Schiller et al. [47] noted higher amygdala activation during the aversive cue but greater striatal and prefrontal activation in the presence of the safety cue and vice-versa when the value of the cues was shifted, indicating that other structures could mediated flexible behavioral modulation. By contrast, in the Human Intruder task, the animals must modulate their emotional reactivity on the basis of a single exposure. Although the animals were familiar with many human beings portraying different emotional signals, in the presence of an unfamiliar human, they must adapt rapidly and their rapid shift in emotional reactivity may rely, as for the reinforcer- devaluation task, on amygdala-orbitofrontal interactions. In summary, the role of the amygdala in flexible modulation of goal-directed behaviors or of emotional reactivity may not rely on the discrimination or representation of positive or negative value of external or internal signals, but rather on rapidly updating the valence of the cues on the span of a single exposure; a function that may be realized only by the direct cross-talks between the amygdala and orbital frontal cortex.

### Role of gender and flexible decision-making

It was also interesting to note that performance of females in the reinforcer devaluation tests tended to be superior to that of males and this gender difference was present in the experimental and control animals alike. Although these findings will need to be replicated with a larger sample size, Overman et al. [48] had already shown that, in both humans and monkeys, gender played a transient role in development of abilities to solve concurrent discriminations and object discrimination reversal learning, with females more efficient than males in the former and males more efficient than females in the latter. However, this gender difference was absent in adult humans or animals. Thus, the presence of gender differences in the adult animals of the current study was unexpected but suggests that the amygdala does not play a critical role in the expression of sexually dimorphic goal-directed behaviors.

### Relationships to clinical disorders

The inflexibility to modulate goal-directed behaviors after neonatal amygdala lesions (present study) as well as after neonatal orbitofrontal lesions [11] parallels results obtained with lesion studies in adult monkeys [49,50]. Both sets of data suggest that the rapid modulation of goal-directed actions required interactions between the amygdala and the orbitofrontal cortex, with the amygdala required to update the positive or negative value of object representations in sensory cortical areas and the orbitofrontal cortex using these updated cortical representations to guide choice selection. Thus, disruption of these interactions may also be the source of behavioral inflexibility and lack of modulation of goal-directed actions reported in several neuropsychiatric disorders associated with amygdala and prefrontal dysfunction, such as Autism Spectrum Disorders [51,52], anxiety disorders including posttraumatic stress disorders [53], and major depressive disorders. For example, the present results strengthen the clinically-driven hypothesis that the rule-learning impairment in ASD is due to the developmental abnormalities in higher-order cortical areas, such as the prefrontal cortex [54] rather than the amygdala. Similarly, growing evidence suggests that the inability to flexibly apply social rules across various situations during behavioral therapy training in people with ASD [55,56] has been attributed to dysfunction of the frontal cortices [57]. Nevertheless, the current findings suggest that dysfunction in amygdala-orbitofrontal interactions in ASD could also result in mental inflexibility. Also, recent clinical studies have begun to examine PTSD using a developmental framework with twin studies or prospective studies examining behavioral flexibility prior to trauma [10]. For example, Admon et al. [58] found that healthy soldiers who would later go on to develop PTSD via their combat service demonstrated imbalanced neural responses in the amygdala and nucleus accumbens when weighing risk versus reward prior to trauma. This neural imbalance suggests that a lack of strong flexible decision-making abilities may have predisposed those individuals to develop PTSD. Thus, the developmental studies in monkeys may help to more tightly define the neural correlates of behavioral dysfunction and provide biomarkers that may be used to better define specific psychiatric disorders, which will lead in turn to more efficacious treatment options.

## Figures and Tables

**Figure 3:**
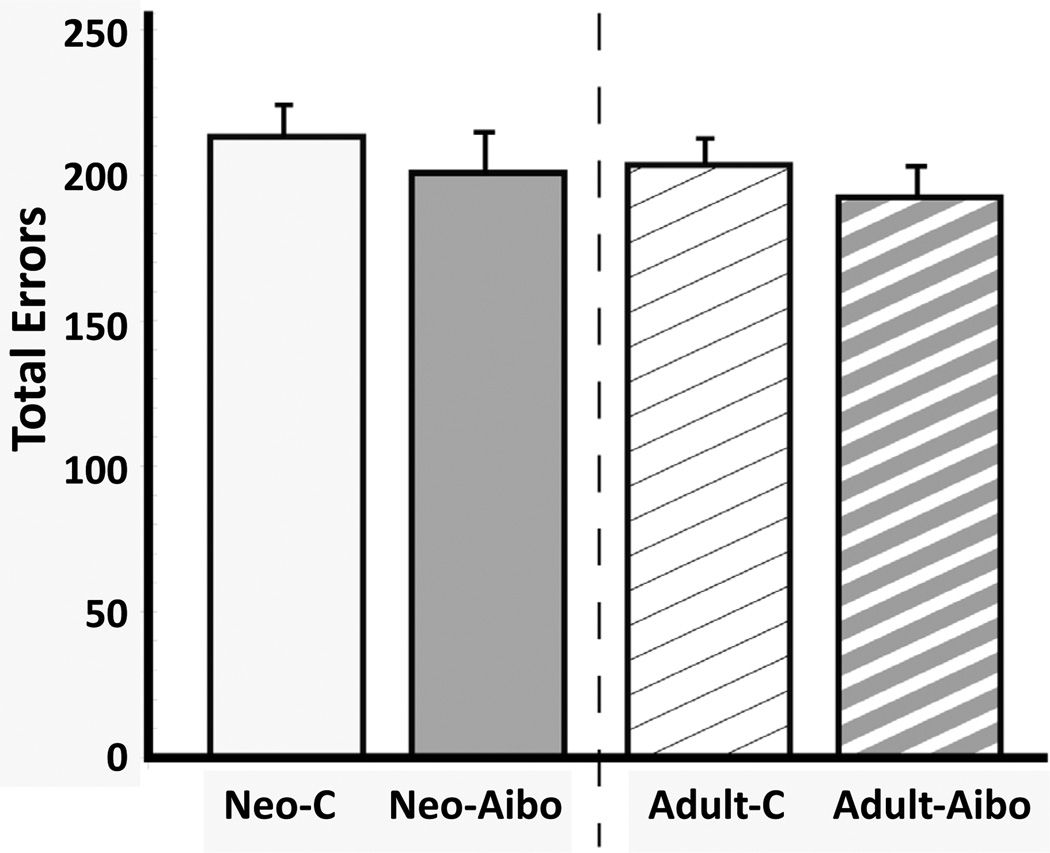
Mean number of errors (± SEM) made before reaching criterion in the concurrent discrimination performance for animals with neonatal lesions (Neo-C and Neo-A-ibo) and for animals with adult-onset lesions (Adult-C and Adult-Aibo; data are from Machado and Bachevalier, 2009) that had learned the task for the first time at the age of approximately 4 years. Criterion was set at 90% correct or better over 5 consecutive days.

**Figure 4:**
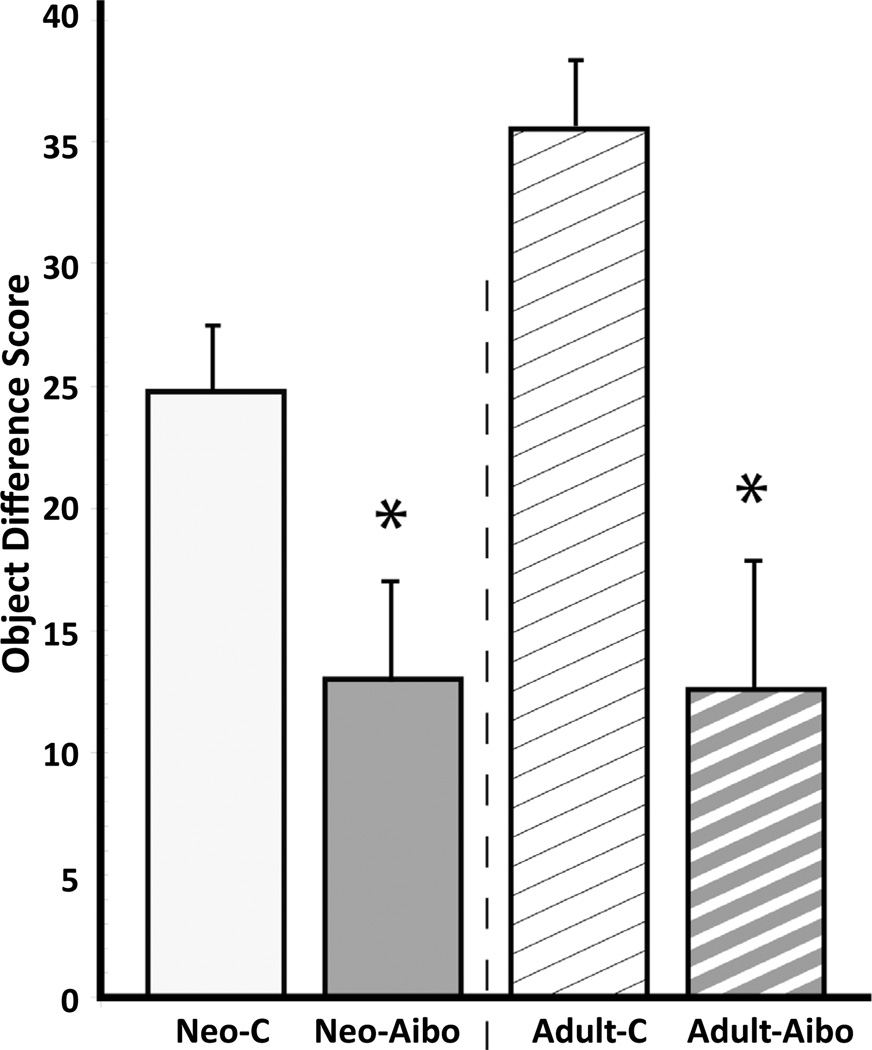
Averaged object difference scores (± SEM) for animals with neonatal lesions (Neo-C and Neo-Aibo) and for animals with adult-onset lesions (Adult-C and Adult-Aibo). Object difference score measures how often a subject chooses an object paired with a devalued food item. *indicates p < 0.05.

**Table 1:** Extent of intended and unintended damage in Group A-ibo

Cases	Amygdala				Hippocampal Formation			
	L	R	Avg	W	L	R	Avg	W
Neo-Aibo-1	89.0	59.8	74.4	53.2	5.1	3.1	4.1	0.2
Neo-Aibo-2	42.0	77.6	59.8	32.6	0	0.8	0.4	0
Neo-Aibo-3	33.0	61.1	47.1	20.2	0	0	0	0
Neo-Aibo-4	62.1	90.0	76.0	55.9	1.9	3.0	2.4	0.1
Neo-Aibo-5	41.2	66.6	53.9	27.5	0	0	0	0
Neo-Aibo-6	52.1	75.6	63.8	39.3	5.6	10.3	8.0	0.6
**X**	**53.2**	**71.8**	**62.5**	**38.1**	**2.1**	**2.9**	**2.5**	**0.1**

Data are the estimated percentage of damage as assessed from MR (post-surgical T1) images. L: percentage of damage to the left hemisphere; R: percentage of damage to the right hemisphere; Avg: average of L and R; W= (L× R)/100X: group mean.

**Table 2:** Cognitive Scores.

	Time at Test		4 yrs			6 yrs	
Sex	Cases	Acq	Object Difference	Food Difference	Retention	Object Difference	Food Difference
	Neo-C						
♀	Neo-C-1	226	21	28	53	26	26
♂	Neo-C-2	180	23	18	0	18	24
♀	Neo-C-3	221	19	30	98	39	29
♂	Neo-C-4	301	22	23	93	17	30
	**X**	**232**	**21.3**	**24.8**	**61**	**25**	**27.3**
	Neo-Aibo						
♀	Neo-Aibo-1	179	22	18	146	11	8
♂	Neo-Aibo-2	313	23	30	12	21	23
♀	Neo-Aibo-3	120	2	27	94	14	26
♂	Neo-Aibo-4	147	6	27	-	-	-
♀	Neo-Aibo-5	262	5	25	9	24	30
♂	Neo-Aibo-6	175	5	5	18	−2	17
	**X**	**199.3**	**10.5**	**22**	**55.8**	**13.6**	**20.8**

Scores are total number of errors made before criterion days for the acquisition (Acq) of the concurrent discrimination task at 4 years of age and retention of the task two years later (6 years). Object difference scores and food difference scores obtained in the devaluation probe sessions at the two ages tested. Neo-C: animals with neonatal sham-operations and Neo-Aibo: animals with neonatal amygdala lesions.

**Table 3:** Satiation Variables

	Time of Test	~4 yrs	~4 yrs	~4 yrs	~6 yrs	~6 yrs	~6 yrs
Sex	Cases	Sat.	Consump.	Weight	Sat.	Consum.	Weight
	**Neo-C**						
♀	Neo-C-1	40	100	7.20	56	70	8.00
♂	Neo-C-2	117	120	7.10	98	200	8.80
♀	Neo-C-3	54	30	9.25	35	40	9.20
♂	Neo-C-4	97	70	7.00	60	100	7.94
	**X**	**77**	**80**	**7.64**	**62.25**	**102.5**	**8.49**
	**Neo-Aibo**						
♀	Neo-Aibo-1	57	110	5.75	69	100	7.20
♂	Neo-Aibo-2	59	90	5.95	38	70	7.80
♀	Neo-Aibo-3	111	105	6.40	38	50	5.60
♂	Neo-Aibo-4	78	40	6.70	-	-	-
♀	Neo-Aibo-5	61	60	6.30	73	60	7.10
♂	Neo-Aibo-6	153	105	8.40	50	95	10.90
	**X**	**86.5**	**85**	**6.58**	**53.6**	**75**	**7.72**

Scores are average time (min) taken for each animal to selectively satiate to the food rewards (Sat), average amount (g) of food eaten (consump.) during all selective satiation sessions, and average weight (Kg) of the animal at the time of the satiation sessions. Other abbreviations as in Table 2.

## References

[1] American Psychiatric Association (2000). Diagnostic and Statistical Manual of Mental Disorders, Fourth Edition: DSM-IV-TR®.

[2] American Psychiatric Association (2013). Diagnostic and statistical manual of mental disorders.

[3] Nesse RM, Stein DJ (2012). Towards a genuinely medical model for psychiatric nosology. BMC Med.

[4] Goh S, Peterson BS (2012). Imaging evidence for disturbances in multiple learning and memory systems in persons with autism spectrum disorders. Dev Med Child Neurol.

[5] Haas BW, Mills D, Yam A, Hoeft F, Bellugi U (2009). Genetic influences on sociability: heightened amygdala reactivity and event-related responses to positive social stimuli in Wlliams syndrome. J Neurosci.

[6] Schumann CM, Bauman MD, Amaral DG (2011). Abnormal structure or function of the amygdala is a common component of neurodevelopmental disorders. Neuropsychologia.

[7] Shepherd AM, Laurens KR, Matheson SL, Carr VJ, Green MJ (2012). Systematic meta-review and quality assessment of the structural brain alterations in schizophrenia. Neurosci Biobehav Rev.

[8] Shin LM, Rauch SL, Pitman RK (2006). Amygdala, medial prefrontal cortex, and hippocampal function in PTSD. Ann N Y Acad Sci.

[9] Del Casale A, Ferracuti S, Rapinesi C, Serata D, Piccirilli M (2012). Functional neuroimaging in specific phobia. Psychiatry Res.

[10] Carrion VG, Kletter H (2012). Posttraumatic stress disorder: shifting toward a developmental framework. Child Adolesc Psychiatr Clin N Am.

[11] Bachevalier J, Machado CJ, Kazama A (2011). Behavioral outcomes of late-onset or early-onset orbital frontal cortex (areas 11/13) lesions in rhesus monkeys. Ann N Y Acad Sci.

[12] Machado CJ, Bachevalier J (2006). The impact of selective amygdala, orbital frontal cortex, or hippocampal formation lesions on established social relationships in rhesus monkeys (Macaca mulatta). Behav Neurosci.

[13] Machado CJ, Bachevalier J (2008). Behavioral and hormonal reactivity to threat: effects of selective amygdala, hippocampal or orbital frontal lesions in monkeys. Psychoneuroendocrinology.

[14] Raper J, Wallen K, Sanchez MM, Stephens SB, Henry A (2013). Sex-dependent role of the amygdala in the development of emotional and neuroendocrine reactivity to threatening stimuli in infant and juvenile rhesus monkeys. Horm Behav.

[15] Raper J, Wilson M, Sanchez M, Machado CJ, Bachevalier J (2013). Pervasive alterations of emotional and neuroendocrine responses to an acute stressor after neonatal amygdala lesions in rhesus monkeys. Psychoneuroendocrinology.

[16] Kazama AM, Heuer E, Davis M, Bachevalier J (2012). Effects of neonatal amygdala lesions on fear learning, conditioned inhibition, and extinction in adult macaques. Behav Neurosci.

[17] Machado CJ, Kazama AM, Bachevalier J (2009). Impact of amygdala, orbital frontal, or hippocampal lesions on threat avoidance and emotional reactivity in nonhuman primates. Emotion.

[18] Kazama AM, Bachevalier J (2012). Preserved stimulus-reward and reversal learning after selective neonatal orbital frontal areas 11/13 or amygdala lesions in monkeys. Dev Cogn Neurosci.

[19] Machado CJ, Bachevalier J (2007). The effects of selective amygdala, orbital frontal cortex or hippocampal formation lesions on reward assessment in nonhuman primates. Eur J Neurosci.

[20] Machado CJ, Bachevalier J (2007). Measuring reward assessment in a semi-naturalistic context: the effects of selective amygdala, orbital frontal or hippocampal lesions. Neuroscience.

[21] Jovanovic T, Kazama A, Bachevalier J, Davis M (2012). Impaired safety signal learning may be a biomarker of PTSD. Neuropharmacology.

[22] Winslow JT, Noble PL, Davis M (2008). AX+/BX- discrimination learning in the fear-potentiated startle paradigm in monkeys. Learn Mem.

[23] Rauch SL, Shin LM, Phelps EA (2006). Neurocircuitry models of posttraumatic stress disorder and extinction: human neuroimaging research—past, present, and future. Biol Psychiatry.

[24] Baxter MG, Murray EA (2002). The amygdala and reward. Nat Rev Neurosci.

[25] Clark L, Cools R, Robbins TW (2004). The neuropsychology of ventral prefrontal cortex: decision-making and reversal learning. Brain Cogn.

[26] Izquierdo A, Murray EA (2007). Selective bilateral amygdala lesions in rhesus monkeys fail to disrupt object reversal learning. J Neurosci.

[27] Rudebeck PH, Murray EA (2008). Amygdala and orbitofrontal cortex lesions differentially influence choices during object reversal learning. J Neurosci.

[28] Izquierdo A, Murray EA (2004). Combined unilateral lesions of the amygdala and orbital prefrontal cortex impair affective processing in rhesus monkeys. J Neurophysiol.

[29] Málková L, Gaffan D, Murray EA (1997). Excitotoxic lesions of the amygdala fail to produce impairment in visual learning for auditory secondary reinforcement but interfere with reinforcer devaluation effects in rhesus monkeys. J Neurosci.

[30] Gottfried JA, O’Doherty J, Dolan RJ (2003). Encoding predictive reward value in human amygdala and orbitofrontal cortex. Science.

[31] Zeeb FD, Wnstanley CA (2013). Functional disconnection of the orbitofrontal cortex and basolateral amygdala impairs acquisition of a rat gambling task and disrupts animals’ ability to alter decision-making behavior after reinforcer devaluation. J Neurosci.

[32] Pickens CL, Saddoris MP, Setlow B, Gallagher M, Holland PC (2003). Different roles for orbitofrontal cortex and basolateral amygdala in a reinforcer devaluation task. J Neurosci.

[33] Baxter MG, Parker A, Lindner CC, Izquierdo AD, Murray EA (2000). Control of response selection by reinforcer value requires interaction of amygdala and orbital prefrontal cortex. J Neurosci.

[34] Bachevalier J, Kazama A, Raper J (2007). Early damage to the amygdala in monkeys alters emotional reactivity, choices guided by reward value but not reward contingency, and social behavior.

[35] Kazama AM, Glavis-Bloom C, Bachevalier J (2008). Neonatal amygdala and orbital frontal cortex lesions disrupt flexible decision-making in adult macaques.

[36] Goursaud AP, Bachevalier J (2007). Social attachment in juvenile monkeys with neonatal lesion of the hippocampus, amygdala and orbital frontal cortex. Behav Brain Res.

[37] Kazama A, Bachevalier J (2009). Selective aspiration or neurotoxic lesions of orbital frontal areas 11 and 13 spared monkeys’ performance on the object discrimination reversal task. J Neurosci.

[38] Payne C, Goursaud AP, Kazama A, Bachevalier J (2007). The effects of neonatal amygdala and orbital frontal lesions on the development of dyadic social interactions in infant rhesus monkeys.

[39] Gellerman LW (1933). Chance Orders of Alternating Stimuli in Visual Discrimination Experiments. The Pedagogical Seminary and Journal of Genetic Psychology.

[40] Bachevalier J, Beauregard M, Alvarado MC (1999). Long-term effects of neonatal damage to the hippocampal formation and amygdaloid complex on object discrimination and object recognition in rhesus monkeys (Macaca mulatta). Behav Neurosci.

[41] Izquierdo A, Suda RK, Murray EA (2004). Bilateral orbital prefrontal cortex lesions in rhesus monkeys disrupt choices guided by both reward value and reward contingency. J Neurosci.

[42] Antoniadis EA, Winslow JT, Davis M, Amaral DG (2007). Role of the primate amygdala in fear-potentiated startle: effects of chronic lesions in the rhesus monkey. J Neurosci.

[43] Davis M (1992). The role of the amygdala in fear-potentiated startle: implications for animal models of anxiety. Trends Pharmacol Sci.

[44] LeDoux JE (2000). Emotion circuits in the brain. Annu Rev Neurosci.

[45] Wellman LL, Gale K, Malkova L (2005). GABAA-mediated inhibition of basolateral amygdala blocks reward devaluation in macaques. J Neurosci.

[46] Rhodes SE, Charles DP, Howland EJ, Murray EA (2012). Amygdala lesions in rhesus monkeys fail to disrupt object choices based on internal context. Behav Neurosci.

[47] Schiller D, Levy I, Niv Y, LeDoux JE, Phelps EA (2008). From fear to safety and back: reversal of fear in the human brain. J Neurosci.

[48] Overman WH, Bachevalier J, Schuhmann E, Ryan P (1996). Cognitive gender differences in very young children parallel biologically based cognitive gender differences in monkeys. Behav Neurosci.

[49] Murray EA, Izquierdo A (2007). Orbitofrontal cortex and amygdala contributions to affect and action in primates. Ann N Y Acad Sci.

[50] Murray EA, Wise SP (2010). Interactions between orbital prefrontal cortex and amygdala: advanced cognition, learned responses and instinctive behaviors. Curr Opin Neurobiol.

[51] Loveland KA, Bachevalier J, Pearson DA, Lane DM (2008). Fronto-limbic functioning in children and adolescents with and without autism. Neuropsychologia.

[52] Edgin JO, Pennington BF (2005). Spatial cognition in autism spectrum disorders: superior, impaired, or just intact?. J Autism Dev Disord.

[53] Neylan TC, Lenoci M, Rothlind J, Metzler TJ, Schuff N (2004). Attention, learning, and memory in posttraumatic stress disorder. J Trauma Stress.

[54] Jones EJ, Webb SJ, Estes A, Dawson G (2013). Rule learning in autism: the role of reward type and social context. Dev Neuropsychol.

[55] Reed P, Watts H, Truzoli R (2013). Flexibility in young people with autism spectrum disorders on a card sort task. Autism.

[56] Barbaro J, Dissanayake C (2007). A comparative study of the use and understanding of self-presentational display rules in children with high functioning autism and Asperger’s disorder. J Autism Dev Disord.

[57] Hill EL (2004). Executive dysfunction in autism. Trends Cogn Sci.

[58] Admon R, Lubin G, Rosenblatt JD, Stern O, Kahn I (2013). Imbalanced neural responsivity to risk and reward indicates stress vulnerability in humans. Cereb Cortex.

